# Raw Equid Milk: A Potential Risk for Q Fever?

**DOI:** 10.3390/ani15101460

**Published:** 2025-05-19

**Authors:** Elisa Mazzotta, Alda Natale, Laura Bellinati, Letizia Ceglie, Laura Lucchese, Tahsin Onur Kevenk, Maria Luisa Menandro, Federica Giacometti, Leonardo Alberghini

**Affiliations:** 1 Istituto Zooprofilattico Sperimentale delle Venezie, 35020 Legnaro, PD, Italy; anatale@izsvenezie.it (A.N.); lbellinati@izsvenezie.it (L.B.); lceglie@izsvenezie.it (L.C.); llucchese@izsvenezie.it (L.L.); 2 Nova Biologicals, 1775 N Loop 336 E, Conroe, TX 77301, USA; tahsin.kevenk@tentamus.com; 3 Department of Animal Medicine, Productions and Health, University of Padova, 35020 Legnaro, PD, Italy; marialuisa.menandro@unipd.it (M.L.M.); federica.giacometti@unipd.it (F.G.); leonardo.alberghini@unipd.it (L.A.)

**Keywords:** *Coxiella burnetii*, equid milk, human consumption, Q fever, zoonosis

## Abstract

Equid milk (donkey or mare) has traditionally been consumed raw or fermented due to its distinctive chemical composition, which closely resembles human milk, and its nutritional benefits. Nowadays, interest in it is increasing, despite limited research on its microbiological risk. *Coxiella burnetii* (*C. burnetii*), a globally re-emerging zoonotic agent, has rarely been investigated in equid milk. The objective of this study was to demonstrate the applicability of selected analytical methods for detecting *C. burnetii* in raw equid milk. A commercial molecular assay was tested on three serial dilutions of one donkey milk sample contaminated with a *C. burnetii* plasmid, which showed no interference of donkey milk with real-time PCR detection. The commercial molecular assay and a serological assay routinely used in the laboratory were also applied to screen a total of 106 equid milks from 16 farms to assess their applicability to diagnostic samples. No *C. burnetii* DNA or anti-*C. burnetii* antibodies were detected in these screenings. In our study, the implemented methods appeared to be suitable for *C. burnetii* investigation in equid milk. While the overall low prevalence recorded in Italy among domestic species is reassuring, strategic surveillance and risk assessment remain essential considering the low infectious dose of *C. burnetii* and its zoonotic potential.

## Simple Summary

Interest in equid milk is growing, although limited research exists on its microbiological risks. *Coxiella burnetii* is a zoonotic pathogen with global concern, but it has been infrequently investigated in equid milk. This study aimed to assess the applicability of analytical methods for detecting *C. burnetii* in raw equid milk using a commercial molecular assay on bulk tank donkey milk. In addition, this study screened 106 equid milk samples from 16 farms using both molecular and serological tests, with no detection of *C. burnetii* DNA or antibodies. Results suggest these methods are effective for *C. burnetii* detection in equid milk. Although the low prevalence in Italy is reassuring, surveillance and risk assessment are crucial.

## 1. Introduction

*Coxiella burnetii* (*C. burnetii*) is a zoonotic obligate intracellular bacterium with a worldwide distribution. It is the causative agent of Q fever in humans and coxiellosis in animals [1,2,3]. This zoonotic agent manifests in humans with a range of symptoms including fever, hepatitis, and pneumonia. It can also lead to chronic infections that can result in endocarditis, vascular infections, osteoarticular infections, and lymphadenitis [2,4,5,6]. While sheep, cattle, and goats are the main carriers of human infection [7,8,9,10,11], and it is considered an occupational zoonotic disease, cases have also been recorded in humans without direct contact with animals [9].

Equid milk has been used since ancient times in cosmetics and as nourishment. Today, its popularity is growing due to its role as functional food for human nutrition in the third millennium. Equid milk possesses functional and bioactive properties, including immunomodulatory and anti-inflammatory effects, attributed to its unique chemical composition [12,13,14]. Particularly, equid milk is characterized by high levels of essential fatty acids (including α-linoleic acid), low levels of saturated fatty acids [15,16], lactose as a primary source of carbohydrates promoting the growth of probiotic microorganisms [17,18], vitamins of groups A, B, C, D, and E, and minerals essential for bone growth and general health [15,19,20]. In addition, due to its lower casein content and reduced casein-to-whey protein ratio compared to cow milk, equid milk is considered a potential alternative for children with a cow’s milk protein allergy. It is also a suitable nutritional option for growing children, convalescent patients, and the elderly [21,22,23].

The estimated contribution of equid milk to global milk production does not exceed 0.5% (https://www.fao.org/dairy-production-products/production/dairy-animals/en/ accessed on 15 January 2025) [24]. Equid milk could be marketed in various forms as pasteurized (or heat-treated) milk, milk powders, or fermented functional drinks, or after specific processing treatments, such as high-pressure processing, to extend its shelf-life and enhance safety [15,24,25]. Conversely, in accordance with tradition, culture, or a deliberate decision to maintain the integrity of its original nutritional components, equid milk is predominantly marketed in its raw state. Although oral infection is not considered to be a primary route of transmission for *C. burnetii*, the analysis of unpasteurized milk is a critical issue that requires attention [5,6,26,27]. Furthermore, the population group most susceptible to infection from *C. burnetii*-contaminated raw milk includes individuals with compromised immune systems [28,29]. This demographic is predominantly composed of children, pregnant women, and the elderly [11,30,31].

It is recognized that the evaluation of raw equid milk should primarily focus on potential microbiological hazards [32,33,34]. Nevertheless, limited information is available on its microbial ecology, and uncertainty remains regarding the sources of infection in children and adults. As a result, authoritative researchers fear that equid milk may become a product requiring closer scrutiny [20,27,35].

As previously reported, microbial contamination and the somatic cell count in equid milk are low, but in regions with a high prevalence of *Brucella* spp. and *Prescottella equi* (formerly *Rhodococcus equi*), the risk of milk infection with this pathogen increases [36]. Recently, *Toxoplasma* (*T.*) *gondii* have been detected in the serum and milk of donkeys in China [37,38] and other countries worldwide, as well in Europe [39,40].

European Union legislation establishes regulatory frameworks for the dairy sector to ensure consumer health protection. These frameworks are built upon several key legislations, including Regulation (EC) No 178/2002 [41], Regulation (EC) No 852/2004 [42], Regulation (EC) No 853/2004 [43], Regulation (EC) No 2017/625 [44], and Regulation (EC) 2019/627 [45], which collectively constitute the legal basis for the production, trade, and official control of food of animal origin. A comprehensive overview of regulations and specific guidance has been detailed by Čapla et al. [46]. Moreover, in the EU, there are no harmonized rules or recommendations for the monitoring and reporting of Q fever in animals. Q fever is not explicitly listed in Annex I to Directive 2003/99/EC of the European Parliament and of the Council on the monitoring of zoonoses and zoonotic agents, amending Council Decision 90/424/EEC and repealing Council Directive 92/117/EEC [3], does not list *C. burnetii* among zoonotic agents requiring epidemiological investigation in foodborne outbreaks [47]. For this reason, despite the existence of official data regarding the hygiene and health parameters of equid milk, the available information concerning the presence of *C. burnetii* is limited. Furthermore, the characterization of raw equid milk as a possible vehicle of *C. burnetii* for humans is also lacking.

So far, a validated protocol for the molecular and serological detection of *C. burnetii* in ruminant milk is currently employed at the Istituto Zooprofilattico Sperimentale delle Venezie. The objective of this study was to demonstrate the applicability of the analytical protocols to fresh raw donkey milk and frozen raw horse milk in order to investigate the presence of *C. burnetii* in the Italian equid population. The aim was to facilitate a standardized, efficient, and practical method for testing raw equid milk for human consumption.

## 2. Materials and Methods

### 2.1. Study Design

A total of 106 equid milks were collected from 16 farms, including 90 individual donkey (*Equus asinus*) raw milk samples from 11 donkey farms, 6 donkey raw bulk tank milks (BTMs) from 4 donkey farms, and 10 horse (*Equus caballus*) raw BTMs from 1 horse farm. Donkey milk is sold raw and fresh in 500 mL or 1 L portions, while horse milk is sold raw and frozen in 250 mL portions. Donkey milk samples were kept refrigerated at +4 °C, while horse milk samples were kept frozen at −20 °C, following routine commercial processing. All samples were transported to the laboratory within 48 h of collection.

### 2.2. Diagnostic Methods Assessment

#### 2.2.1. qPCR Analyses of Bulk Tank Milk: Applicability Test

To evaluate the applicability of a real-time PCR (qPCR) protocol to equid BTM, one negative donkey BTM sample was first diluted (1:3) in the inactivating transport medium PrimeStore^®^ (Longhorn Vaccines and Diagnostics, Bethesda, MD, USA). A commercial quantified *C. burnetii* plasmid provided with the qPCR kit ID Gene™ Q Fever Triplex (IDvet, Grabels, France) was then added to a 1:10 dilution. Two additional serial dilutions (1:100 and 1:1000) of the contaminated milk were subsequently prepared using negative milk. The plasmid was quantified at 5 × 10^6^ equivalent genome/mL (EG/mL) by the producer (Idvet, Grabels, France) using the reference DNA (code AND *C. burnetii* NM) from the French National Reference Laboratory (ANSES Sophia-Antipolis, France). The three serial dilutions of the raw milk sample contaminated with the commercial plasmid were tested in triplicate pre- and post-dilution.

Bacterial DNA was extracted from contaminated milk samples using the ID Gene^®®^ Mag Universal Extraction Kit (Idvet, Grabels, France), in accordance with the manufacturer’s instructions, on the King Fisher Flex 96 (Thermo Fisher, Waltham, MA, USA). A sample of the undiluted milk was extracted contextually as a negative process control.

The presence of *C. burnetii* DNA was assessed using the commercial real-time PCR (qPCR) kit, the ID Gene™ Q Fever Triplex (Idvet, Grabels, France), targeting the transposon-like IS1111 repetitive region of *C. burnetii* [48], on the QuantStudio™ 5 thermal cycler (Thermo Fisher Scientific, Waltham, MA, USA). The commercial kit included an exogenous internal control, which was co-extracted and co-amplified to validate each negative result. Moreover, each amplification run included water as a negative control and a DNA sample positive to *C. burnetii* provided with the qPCR kit as a positive control. The commercial qPCR kit used has been validated in our laboratory for detecting *C. burnetii* DNA in ruminant milk, and its limit of detection (LoD) for this type of specimen during the validation process was determined to be 300 GE/mL.

#### 2.2.2. qPCR Analyses of Individual Milk Samples

Ninety individual raw milk samples were collected from donkeys at 11 Italian farms from January to June 2010, during a single milk production cycle. Samples were stored at the laboratories of the Istituto Zooprofilattico Sperimentale delle Venezie at ≤−70 °C until further analysis.

DNA was extracted from 400 µL of milk using the QIAamp DNA mini kit (QIAGEN, Hilden, Germany) according to the manufacturer’s instructions. Samples were screened for the presence of *C. burnetii* by the commercial qPCR kit ADIAVET™ COX REAL TIME (ADIAVET, Rochefort, Belgium), targeting the transposon-like IS1111 repetitive element, on the 7900HT Fast Real-Time PCR System thermal cycler (Thermo Fisher Scientific, Waltham, MA, USA). The contextual amplification of an endogenous internal control validated each negative result. Each amplification run included water as a negative control and a DNA sample positive to *C. burnetii* provided with the qPCR kit as a positive control.

#### 2.2.3. qPCR Analyses of Bulk Tank Milk Samples

The 16 BTM samples tested were purchased directly from producing farms between 2022 and 2024, and stored frozen at ≤−200 °C. Upon arrival at the laboratory, all samples were stored at ≤−70 °C until further analysis.

After thawing and vortexing, DNA was extracted from 100 µL of milk and tested with the commercial qPCR kit ID Gene™ Q Fever Triplex (Idvet, Grabels, France), as described in Section 2.2.1. Each DNA extraction included a negative control (water). The exogenous internal control supplied in the commercial kit was co-extracted and co-amplified to validate each negative result. Every amplification run included water as a negative control and a DNA sample positive to *C. burnetii* provided with the qPCR kit as a positive control.

#### 2.2.4. Serological Investigation: ELISA Assay of Milk Samples

The commercial indirect ELISA assay ID Screen^®^ Q Fever Indirect Multi-species (Idvet, Grabels, France) was used to detect antibodies directed against *C. burnetii* in all milk samples. This kit contained a multispecies conjugate already validated for equids [49,50,51].

In accordance with the manufacturer’s instructions, whole milk samples were left to sit to allow cream separation. The resulting lactoserum was diluted 1:50 in the provided dilution buffer, aliquoted into the ELISA microplate coated with Phase 1 and Phase 2 *C. burnetii* antigens, and incubated at room temperature (21 °C ± 5 °C). After washing, an anti-multi-species conjugate was added to each well and incubated for 30 min at 21 °C ± 5 °C, followed by washing. The substrate solution was then added, and after an incubation period in the dark, the stop solution was added to each well. An optical density (OD) at 405 nm has been reported. After verifying the validity criteria for positive and negative controls, the sample/positive OD percentage (S/P%) was calculated for each sample. Individual milks were considered negative with an S/P% ≤ 40%, doubtful with an S/P% between 40% and 50%, and positive with an S/P% over 80%. Meanwhile, BTM samples were considered negative with an S/P% ≤ 30%, doubtful with an S/P% between 30% and 40%, and positive with an S/P% over 40%.

## 3. Results

### 3.1. Samples Distribution

**Figure 1:**
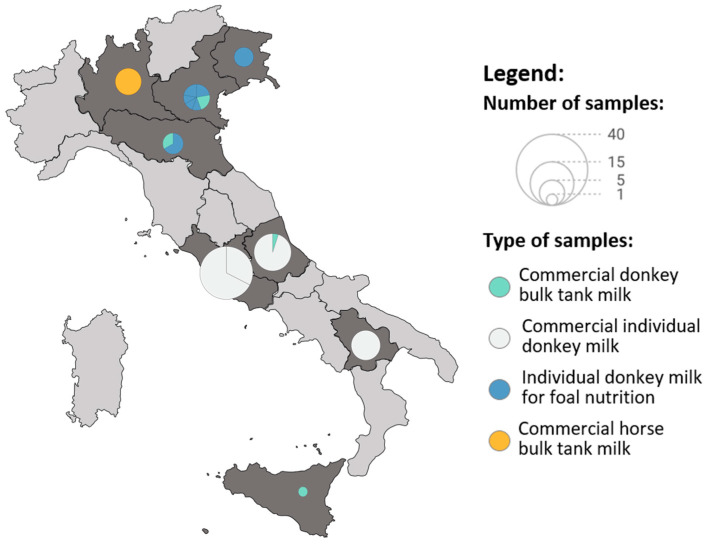
Samples’ distribution. Each circular area depicts the selected farms within the Italian region, with its size indicating the number of samples collected. The colors of the circles refer to the type of sample: commercial bulk donkey milk, individual donkey milk, individual donkey milk for foal nutrition, and commercial bulk mare milk.

In this study, a total of 106 equid milks from 16 Italian farms were tested for the presence of *C. burnetii* DNA and anti-*C. burnetii* antibodies. Figure 1 shows the location of these farms in Italy; 10 are located in northern Italy, 4 in the central region, and 2 in the South of Italy. Location, breed, type of feeding, whether the milk was used for foal feeding only or for commercial purposes (e.g., for human consumption or in the pharmaceutical, medical, or dermo-cosmetic sectors), whether the farm kept animals for pet therapy, and details regarding sample type (individual or BTM) and milking methods are specified in Table 1.

At farms N and O, BTM was collected in two consecutive years (from 2023 to 2024), and in farm P 10 milks from the mare were collected at different times during one month of the production period of the same year (2024).

Seven of the farms were amateur, small to medium-sized with under 10 animals, and their milk was used for foal nutrition. The other nine farms housed over 10 animals and were dedicated to donkey breeding or the production of donkey milk for human consumption, cosmetic products, or medical purposes. The information collected for each farm is summarized in Table 1.

### 3.2. Molecular Method Assessment

The sample of raw donkey BTM was tested by qPCR after a first dilution (1:3) in the inactivating transport medium, and had a negative result. After qPCR analyses on three serial raw milk dilutions contaminated with the *C. burnetii* commercial plasmid, tested in triplicate pre- and post-dilution, the average cycle threshold (Ct) obtained was compared to the expected Ct of the *C. burnetii* plasmid indicated by the manufacturer. No interference of donkey milk, except for one Ct shift, was observed in these qPCR analyses, as depicted in Table 2.

### 3.3. Diagnostic Investigation of Raw Equid Milk

A total of 106 equid milks from 16 Italian farms were tested for the presence of *C. burnetii* DNA and anti-*C. burnetii* antibodies, and all had negative results as reported in Table 3.

## 4. Discussion

Data on the prevalence of *C. burnetii* in equids are limited and often influenced by variability in sampling, testing, and analytical methods used. Indeed, surveillance for Q fever is often implemented in equids only following outbreaks in humans [31,52].

In equids, the *C. burnetii* infection can present as either an asymptomatic condition or a clinical disease [30,53,54,55]. *C. burnetii* infections in non-pregnant horses include reported fever, conjunctivitis, and respiratory and gastrointestinal symptoms [55]. Abortion, premature delivery, and stillbirth have been associated with *C. burnetii* natural infections in equids [54,56]. Nevertheless, the definition of equids as hosts, reservoirs, or sentinel species of this disease and their epidemiological role are still unclear [57]. So far, little information about the epidemiology of *C. burnetii* in equids has been available. Marenzoni et al., in 2013 [54], indicated a possible role of horses as sources of the pathogen for other animal species, as well as humans. Similarly, other studies stated that horses are probably reservoirs of *C. burnetii* for other susceptible animals [10,54,58]. The most recent data on the prevalence of *C. burnetii* infection in horses in the European Union and worldwide report an anti-*C. burnetii* antibody positivity of 2.17% in Slovakia (2022) [57], the molecular detection of *C. burnetii* in 7.50% of horse sera in Iran (2020) [55], and a seroprevalence of 5.64% in 2021 [59]. In addition, anti-*C. burnetii* antibody positivities of 9.9% in Algeria (2018) [60] and 0% in Poland (2017) [61] have been reported. More recently, a *C. burnetii* molecular detection positivity of 0.7% and seroprevalence of 1.3% in South Korea (2016) [58], as well as a seroprevalence of 4% to 12% between 2015 and 2016 in an endemic area in France [62], have been documented. Recently, a relevant seroprevalence (79.2%) in cattle and dairy herds in Denmark (2014) have been described [63,64]. To the best of these authors’ knowledge, no recent serological studies have been conducted in Italy on equid milk to investigate the prevalence of *C. burnetii*.

However, it is crucial to contextualize these values by considering the sampling method, which often involves passive or syndromic surveillance, and the limited number of animals sampled. Furthermore, surveillance data for horses, donkeys, and raw equid milk for human consumption are even less complete [54].

Milk can be contaminated by pathogens either directly shed from the udder or through various environmental sources during or after milking. Potential contamination sources include the teat apex, milking equipment, air, water, feed, grass, soil, and other environmental factors [33,65]. The nutrient-rich, neutral pH of raw equid milk creates ideal conditions for pathogenic microorganisms to survive and grow [36,66].

In addition to the direct use of raw equid milk, different milk derivatives are produced, such as the probiotic drink known as Kumis, cheese, ice cream, powdered milk, and sweets. This highlights the need for greater awareness within the food industry and equid farms regarding food safety, particularly in countries in which the disease has been reported as highly prevalent among domestic and wild species [67,68,69]. Implementing analytical tools for the surveillance of foodborne zoonotic diseases is essential to ensure public health protection [15,70,71]. Furthermore, prospecting a wide dissemination of this innovative food, especially in the context of feeding infants, the immunocompromised, pregnant women, and the elderly, requires a deeper understanding of raw equid milk safety prerequisites [36].

The European Union has recently articulated its position on the necessity of harmonizing the surveillance of specific zoonotic pathogens from a One Health perspective. Among the identified 10 priority zoonotic diseases, it encompasses the management of Q fever [72]. Moreover, the European Food Safety Agency (EFSA) has published a series of guidance documents, opinions, and other reports in an attempt to elucidate the microbiological hazards and public health risks associated with the consumption of raw milk and dairy products [65,73]. While the scientific literature contains a wealth of guidance and monitoring plans for bovine, buffalo, goat, and sheep’s milk, there is still no harmonized plan to approach and identify the risk of Q fever from the consumption of raw equid milk foods. The heterogeneity of assays employed for the evaluation of *C. burnetii* positivity in equids, the absence of standardization in monitoring plans, and the scarcity of tests for direct detection in food matrices (e.g., equid milk) constitute a significant challenge [74].

Our study demonstrated the applicability of a qPCR method to detect *C. burnetii* DNA in raw equid milk, and reported no interference of the matrix with the sensitivity of this method. Thus, equid BTM sampling could be suggested as a *C. burnetii* potential monitoring tool, according to the EU’s One Health Zoonoses Report 2023 [70], in which BTM sampling is reported to be increasingly used as a method for monitoring *C. burnetii* in dairy herds. Moreover, with regard to its use in relation to the herd size and the number of lactating animals, analyses performed in our study on equid BTM diluted and contaminated samples showed the almost equivalent sensitivity of this test (500 GE/mL) as it was assessed during the validation process with ruminant BTM (300 GE/mL). To evaluate the applicability to diagnostic samples, two commercial assays routinely used in the laboratory, one molecular and one serological, were applied to screen a total of 106 equid milks originating from 16 equid farms. No *C. burnetii* DNA or anti-*C. burnetii* antibodies were detected during screening, which is consistent with the low prevalence reported in previous studies in Italy [4,75]. However, questions remain open about screening sensitivity, particularly in relation to herd size and the number of lactating females, raising concerns about its overall efficacy as a surveillance tool [73].

In light of the re-emergence of Q fever and coxiellosis in numerous countries [71] and their potential association with climate change [76], the implementation of surveillance measures in the context of equid milk intended for human consumption is of the utmost importance.

## 5. Conclusions

*Coxiella burnetii* is a zoonotic agent re-emergent worldwide, which is rarely investigated in equid milk.

In our study, the implemented molecular and serological methods appeared to be suitable for the investigation of *C. burnetii* in equid milk and detected no positivity in Italian commercial milk samples. The overall low prevalence recorded in Italy among domestic species is encouraging, but strategic surveillance and risk assessment for operators and consumers are crucial considering the low infectious dose of *C. burnetii* and its zoonotic potential.

## Figures and Tables

**Table 1:** Epidemiological, geographic, and management data of collected samples.

Farm	Region	Equids	Herd Breed	Feeding	Pet Therapy	Milk Usage	Milk Sample Type	Milking Practice	N° of Samples
A	Veneto	Donkey	Crossbreed	Grazing/Hay	No	Foal nutrition	Individual	By hand	2
B	Veneto	Donkey	Crossbreed	Grazing/Hay	Yes	Foal nutrition	Individual	By hand	2
C	Veneto	Donkey	Martina Franca	Grazing/Hay	No	Foal nutrition	Individual	By hand	1
D	Veneto	Donkey	Crossbreed	Grazing/Hay	No	Foal nutrition	Individual	By hand	1
E	Veneto	Donkey	Crossbreed	Grazing/Hay	No	Foal nutrition	Individual	By hand	1
F	Friuli-Venezia Giulia	Donkey	Crossbreed	Grazing/Hay + Protein supplement	No	Foal nutrition	Individual	By hand	5
G	Emilia-Romagna	Donkey	Crossbreed	Grazing/Hay + Protein supplement	Yes	Foal nutrition	Individual	By hand	4
H	Basilicata	Donkey	Crossbreed	Grazing/Hay + Protein supplement	No	Commercial	Individual	Automatic in a milking room	12
I	Lazio	Donkey	Crossbreed	Grazing/Hay + Protein supplement	No	Commercial	Individual	With a portable milker	14
L	Lazio	Donkey	Crossbreed	Grazing/Hay + Protein supplement	No	Commercial	Individual	Automatic in a milking room	29
M	Abruzzo	Donkey	Crossbreed	Grazing/Hay	No	Commercial	Individual	Automatic in a milking room	19
N	Veneto	Donkey	Crossbreed	Grazing/Hay	No	Commercial	BTM	Automatic in a milking room	2
O	Emilia-Romagna	Donkey	Crossbreed	Grazing/Hay	No	Commercial	BTM	Automatic in a milking room	2
P	Lombardia	Horse	Haflinger	Grazing/Hay	No	Commercial	BTM	Automatic in a milking room	10
Q	Abruzzo	Donkey	Crossbreed	Grazing/Hay	No	Commercial	BTM	Automatic in a milking room	1
R	Sicilia	Donkey	Crossbreed	Grazing/Hay	No	Commercial	BTM	Automatic in a milking room	1
Total									106

BTM: bulk tank milk.

**Table 2:** Results of the qPCR method assessment.

Sample Dilution	qPCR Results Pre-Spike	Plasmid Dilution (EG/mL)	Expected Result	qPCR Results Post-Spike
1:10	Negative	5 × 10^4^	Ct 29.32	Ct 30.17
1:100	Negative	5 × 10^3^	Ct 33.22	Ct 32.53
1:1000	Negative	5 × 10^2^	Ct 35.08	Ct 36.01

**Table 3:** Results of molecular investigation of equid milk samples.

Farm	N° of Samples	Result *C. burnetii* Molecular Detection	Result Anti-*C. burnetii* Antibodies
A	2	Negative	Negative
B	2	Negative	Negative
C	1	Negative	Negative
D	1	Negative	Negative
E	1	Negative	Negative
F	5	Negative	Negative
G	4	Negative	Negative
H	12	Negative	Negative
I	14	Negative	Negative
L	29	Negative	Negative
M	19	Negative	Negative
N	2	Negative	Negative
O	2	Negative	Negative
P	10	Negative	Negative
Q	1	Negative	Negative
R	1	Negative	Negative
Total	106		

## Data Availability

Metadata are available from the authors following a reasonable request. Preliminary data have been presented as conference paper (poster presentation): Bellinati L., Ceglie L., Kevenk T.O., Lucchese L., Natale A., Giacometti F., Alberghini L. Investigation on Q Fever agent in donkey milk. Conference paper in EAVLD 2024—7th Congress of the European Association of Veterinary Laboratory Diagnosticians, Padova, PD, Italia, 21–23 October 2024, pp. 294–295 [77].

## References

[1] Celina S.S., Cerný J. (2022). *Coxiella burnetii* in Ticks, Livestock, Pets and Wildlife: A Mini-Review. Front. Vet. Sci..

[2] Eldin C., Mélenotte C., Mediannikov O., Ghigo E., Million M., Edouard S., Mege J.-L.L., Maurin M., Raoult D. (2017). From Q Fever to *Coxiella burnetii* Infection: A Paradigm Change. Clin. Microbiol. Rev..

[3] EFSA Panel on Animal Health and Welfare (AHAW) (2010). Scientific Opinion on QFever. EFSA J..

[4] Alessiani A., di Domenico M., Averaimo D., Pompilii C., Rulli M., Cocco A., Lomellini L., Coccaro A., Cantelmi M.C., Merola C. (2024). *Coxiella burnetii*: A Brief Summary of the Last Five Years of Its Presence in the Abruzzo and Molise Regions in Italy. Animals.

[5] Khademi P., Ownagh A., Ataei B., Kazemnia A., Enferadi A., Khalili M., Mardani K. (2020). Prevalence of C. Burnetii DNA in Sheep and Goats Milk in the Northwest of Iran. Int. J. Food Microbiol..

[6] Raoult D., Marrie T., Mege J. (2005). Natural History and Pathophysiology of Q Fever. Lancet Infect. Dis..

[7] Körner S., Makert G.R., Ulbert S., Pfeffer M., Mertens-Scholz K. (2021). The Prevalence of *Coxiella burnetii* in Hard Ticks in Europe and Their Role in Q Fever Transmission Revisited-A Systematic Review. Front. Vet. Sci..

[8] Melenotte C., Million M., Raoult D. (2020). New Insights in *Coxiella burnetii* Infection: Diagnosis and Therapeutic Update. Expert Rev. Anti. Infect. Ther..

[9] Christodoulou M., Malli F., Tsaras K., Billinis C., Papagiannis D. (2023). A Narrative Review of Q Fever in Europe. Cureus.

[10] Roest H.I.J., Bossers A., van Zijderveld F.G., Rebel J.M.L. (2013). Clinical Microbiology of *Coxiella burnetii* and Relevant Aspects for the Diagnosis and Control of the Zoonotic Disease Q Fever. Vet. Q..

[11] Meles D., Khairullah A., Mustofa I., Wurlina W., Akintunde A., Suwasanti N., Mustofa R., Putra S., Moses I., Kusala M. (2024). Navigating Q Fever: Current Perspectives and Challenges in Outbreak Preparedness. Open Vet. J..

[12] Vincenzetti S., Pucciarelli S., Polzonetti V., Polidori P. (2017). Role of Proteins and of Some Bioactive Peptides on the Nutritional Quality of Donkey Milk and Their Impact on Human Health. Beverages.

[13] Salimei E., Fantuz F. (2012). Equid Milk for Human Consumption. Int. Dairy J..

[14] Uniacke-Lowe T., Fox P.F. (2022). Equid Milk. Encyclopedia of Dairy Sciences.

[15] Papademas P., Mousikos P., Aspri M. (2022). Valorization of Donkey Milk: Technology, Functionality, and Future Prospects. JDS Commun..

[16] Doreau M., Martin-Rosset W. (2011). Animals That Produce Dairy Foods: Horse. Encyclopedia of Dairy Sciences.

[17] Bhardwaj A., Pal Y., Legha R.A., Sharma P., Nayan V., Kumar S., Tripathi H., Tripathi B.N. (2020). Donkey Milk Composition and Its Therapeutic Applications. Indian J. Anim. Sci..

[18] Claeys W.L., Verraes C., Cardoen S., De Block J., Huyghebaert A., Raes K., Dewettinck K., Herman L. (2014). Consumption of Raw or Heated Milk from Different Species: An Evaluation of the Nutritional and Potential Health Benefits. Food Control.

[19] Musaev A., Sadykova S., Anambayeva A., Saizhanova M., Balkanay G., Kolbaev M. (2021). Mare’s Milk: Composition, Properties, and Application in Medicine. Arch. Razi Inst..

[20] Hazeleger W.C., Beumer R.R. (2016). Microbial Quality of Raw Horse Milk. Int. Dairy J..

[21] Zhou M., Huang F., Du X., Wang C., Liu G. (2023). Microbial Quality of Donkey Milk during Lactation Stages. Foods.

[22] Khan M.Z., Chen W., Li M., Ren W., Huang B., Kou X., Ullah Q., Wei L., Wang T., Khan A. (2024). Is There Sufficient Evidence to Support the Health Benefits of Including Donkey Milk in the Diet?. Front. Nutr..

[23] Lajnaf R., Feki S., Ben Ameur S., Attia H., Kammoun T., Ayadi M.A., Masmoudi H. (2023). Recent Advances in Selective Allergies to Mammalian Milk Proteins Not Associated with Cow’s Milk Proteins Allergy. Food Chem. Toxicol..

[24] Papademas P., Neokleous I., Mousikos P. (2023). Thermal Processing of Equine Milk—A Review. Int. Dairy J..

[25] Uniacke-Lowe T., Huppertz T., Fox P.F. (2010). Equine Milk Proteins: Chemistry, Structure and Nutritional Significance. Int. Dairy J..

[26] Cho H.-C., Hwang S., Kim E.-M., Park Y.-J., Shin S.-U., Jang D.-H., Chae J.-S., Choi K.-S. (2021). Prevalence and Molecular Characterization of *Coxiella burnetii* in Cattle, Goats, and Horses in the Republic of Korea. Vector-Borne Zoonotic Dis..

[27] Conte F., Panebianco A. (2019). Potential Hazards Associated with Raw Donkey Milk Consumption: A Review. Int. J. Food Sci..

[28] Pexara A., Solomakos N., Govaris A. (2018). Q Fever and Prevalence of *Coxiella burnetii* in Milk. Trends Food Sci. Technol..

[29] Bartelink A.K., Stevens H., van Kregten E., Meijer J.G., Beeres M.P., van Deuren M. (2000). Acute and Chronic Q Fever; Epidemiology, Symptoms, Diagnosis and Therapy of Infection Caused by *Coxiella burnetii*. Ned. Tijdschr. Geneeskd..

[30] Baseri N., Omidi A.H., Latifian M., Mostafavi E., Khademvatan S., Omidifar N., Tabaei S.j.S., Jafari R., Zeinali S., Ghasemi A. (2024). Molecular Examination for *Coxiella burnetii* and Brucella Spp. Infections in Iranian Women Experiencing Spontaneous Miscarriage. BMC Infect. Dis..

[31] Ullah Q., Jamil T., Saqib M., Iqbal M., Neubauer H. (2022). Q Fever—A Neglected Zoonosis. Microorganisms.

[32] Mottola A., Alberghini L., Giaccone V., Marchetti P., Tantillo G., di Pinto A. (2018). Microbiological Safety and Quality of Italian Donkey Milk. J. Food Saf..

[33] Koutsoumanis K., Allende A., Alvarez-Ordóñez A., Bolton D., Bover-Cid S., Chemaly M., Davies R., de Cesare A., Herman L., Nauta M. (2020). Guidance on Date Marking and Related Food Information: Part 1 (Date Marking). EFSA J..

[34] Verraes C., Claeys W., Cardoen S., Daube G., de Zutter L., Imberechts H., Dierick K., Herman L. (2014). A Review of the Microbiological Hazards of Raw Milk from Animal Species Other than Cows. Int. Dairy J..

[35] Conte F., Passantino A. (2007). Isolation of Enterobacter Sakazakii from Ass’ Milk in Sicily: Case Report, Safety and Legal Issues. Travel Med. Infect. Dis..

[36] Colavita G., Amadoro C., Rossi F., Fantuz F., Salimei E. (2016). Hygienic Characteristics and Microbiological Hazard Identification in Horse and Donkey Raw Milk. Vet. Ital..

[37] Chen L., Zhao Z.-J., Meng Q.-F. (2021). Detection of Specific IgG-Antibodies Against Toxoplasma Gondii in the Serum and Milk of Domestic Donkeys During Lactation in China: A Potential Public Health Concern. Front. Cell. Infect. Microbiol..

[38] Liang W., Zhao S., Wang N., Tang Z., Zhao F., Liu M., Jin W., Meng Y., Jia L. (2022). Molecular Occurrence and Risk Factors for Toxoplasma Gondii Infection in Equids in Jilin, China. Sci. Rep..

[39] Dubey J.P., Murata F.H.A., Cerqueira-Cézar C.K., Kwok O.C.H. (2020). Toxoplasma Gondii Infections in Horses, Donkeys, and Other Equids: The Last Decade. Res. Vet. Sci..

[40] Cano-Terriza D., Franco J.J., Jose-Cunilleras E., Buono F., Almería S., Veneziano V., Alguacil E., García J., Villena I., Dubey J.P. (2023). Seroepidemiological Study of Toxoplasma Gondii in Equids in Different European Countries. Zoonoses Public Health.

[41] European Union (2002). Regulation (EC) No 178/2002 of the European Parliament and of the Council of 28 January 2002 Layingdown the General Principles and Requirements of Food Law, Establishing the European Food Safety Authorityand Laying down Procedures in Matters of Food Safet.

[42] European Union (2004). Regulation (EC) No 852/2004 of the European Parliament and of the Council of 29 April 2004 on the Hygieneof Foodstuffs.

[43] European Union (2004). Regulation (EC) No 853/2004 of the European Parliament and of the Council of 29 April 2004 Laying Downspecific Hygiene Rules for Food of Animal Origin.

[44] Menditto A., Anniballi F., Auricchio B., de Medici D.D., Stacchini P. (2017). Regulation (EU) 2017/625 and the “Union Agri-Food Chain Legislation”: A New Comprehensive Approach in Line with the “One Health” Paradigm?. Eur. Food Feed Law Rev..

[45] European Union (2019). Commission Implementing Regulation (EU) 2019/627 of 15 March 2019 Laying down Uniform Practicalarrangements for the Performance of Official Controls on Products of Animal Origin Intended for Humanconsumption in Accordance with Regulation (EU) 2017/625 Of.

[46] Čapla J., Zajác P., Ševcová K., Čurlej J., Fikselová M. (2023). Overview of the Milk and Dairy Products Legislation in the European Union. Legestic.

[47] European Union (2003). Directive 2003/ 99/ EC of the European Parliament and of the Council of 12 December 2003 on the Monitoring of Zoonoses and Zoonotic Agents, Amending Decision 90/ 424/ EEC and Repealing Council Directive 92/ 117/ EEC.

[48] World Organization for Animal Health (WOAH) (2018). Q Fever.

[49] Paştiu A.I., Gyorke A., Villena I., Balea A., Niculae M., Pall E., Spînu M., Cozma V. (2015). Comparative Assessment of Toxoplasma Gondii Infection Prevalence in Romania Using 3 Serological Methods. Bull. Univ. Agric. Sci. Vet. Med. Cluj-Napoca. Vet. Med..

[50] Paştiu A.I., Györke A., Kalmár Z., Bolfă P., Rosenthal B.M., Oltean M., Villena I., Spînu M., Cozma V. (2015). Toxoplasma Gondii in Horse Meat Intended for Human Consumption in Romania. Vet. Parasitol..

[51] Lee S.-H., Lee S.-E., Seo M.-G., Goo Y.-K., Cho K.-H., Cho G.-J., Kwon O.-D., Kwak D., Lee W.-J. (2014). Evidence of Toxoplasma Gondii Exposure among Horses in Korea. J. Vet. Med. Sci..

[52] .Honarmand H. (2012). Q Fever: An Old but Still a Poorly Understood Disease. Interdiscip. Perspect. Infect. Dis..

[53] van der Kolk J.H., Veldhuis Kroeze E.J.B. (2022). Infectious Diseases of the Horse.

[54] Marenzoni M.L., Stefanetti V., Papa P., Casagrande Proietti P., Bietta A., Coletti M., Passamonti F., Henning K. (2013). Is the Horse a Reservoir or an Indicator of *Coxiella burnetii* Infection? Systematic Review and Biomolecular Investigation. Vet. Microbiol..

[55] Khademi P., Ownagh A., Ataei B., Kazemnia A., Eydi J., Khalili M., Mahzounieh M., Mardani K. (2020). Molecular Detection of *Coxiella burnetii* in Horse Sera in Iran. Comp. Immunol. Microbiol. Infect. Dis..

[56] Leon A., Richard E., Fortier C., Laugier C., Fortier G., Pronost S. (2012). Molecular Detection of *Coxiella burnetii* and Neospora Caninum in Equine Aborted Foetuses and Neonates. Prev. Vet. Med..

[57] Drážovská M., Prokeš M., Vojtek B., Mojžišová J., Ondrejková A., Korytár Ľ. (2022). First Serological Record of *Coxiella burnetii* Infection in the Equine Population of Slovakia. Biologia.

[58] Seo M.-G., Lee S.-H., VanBik D., Ouh I.-O., Yun S.-H., Choi E., Park Y.-S., Lee S.-E., Kim J.W., Cho G.-J. (2016). Detection and Genotyping of *Coxiella burnetii* and Coxiella-Like Bacteria in Horses in South Korea. PLoS ONE.

[59] Jaferi M., Mozaffari A., Jajarmi M., Imani M., Khalili M. (2021). Serologic and Molecular Survey of Horses to *Coxiella burnetii* in East of Iran a Highly Endemic Area. Comp. Immunol. Microbiol. Infect. Dis..

[60] Ansel S., Benfodil K., Cherif A.M., Abdelli A., Kaidi R., Miroud K., Ait-Oudhia K. (2020). *Coxiella burnetii* in Horses of Algeria: Seroprevalence and Associated Risk Factors. World’s Vet. J..

[61] Szymańska-Czerwińska M., Jodełko A., Pluta M., Kowalik S., Niemczuk K. (2017). Seroprevalence of *Coxiella burnetii* among Domestic Ruminants and Horses in Poland. Acta Virol..

[62] Desjardins I., Joulié A., Pradier S., Lecollinet S., Beck C., Vial L., Dufour P., Gasqui P., Legrand L., Edouard S. (2018). Seroprevalence of Horses to *Coxiella burnetii* in an Q Fever Endemic Area. Vet. Microbiol..

[63] Agger J.F., Paul S. (2014). Increasing Prevalence of *Coxiella burnetii* Seropositive Danish Dairy Cattle Herds. Acta Vet. Scand..

[64] Agger J.F., Christoffersen A.-B., Rattenborg E., Nielsen J., Agerholm J.S. (2010). Prevalence of *Coxiella burnetii* Antibodies in Danish Dairy Herds. Acta Vet. Scand..

[65] EFSA Panel on Biological Hazards (BIOHAZ) (2015). Scientific Opinion on the Public Health Risks Related to the Consumption of Raw Drinking Milk. EFSA J..

[66] Leedom J.M. (2006). Milk of Nonhuman Origin and Infectious Diseases in Humans. Clin. Infect. Dis..

[67] El-Mahallawy H.S., Kelly P., Zhang J., Yang Y., Zhang H., Wei L., Mao Y., Yang Z., Zhang Z., Fan W. (2016). High Seroprevalence of *Coxiella burnetii* in Dairy Cattle in China. Trop. Anim. Health Prod..

[68] de França D.A., de Souza Ribeiro Mioni M., Fornazari F., de Lima Duré A.Í., Silva M.V.F., Possebon F.S., Richini-Pereira V.B., Langoni H., Megid J. (2022). Seropositivity for *Coxiella burnetii* in Suspected Patients with Dengue in São Paulo State, Brazil. PLoS Negl. Trop. Dis..

[69] Eldin C., Mahamat A., Demar M., Abboud P., Djossou F., Raoult D. (2014). Q Fever in French Guiana. Am. Soc. Trop. Med. Hyg..

[70] Meena S., Meena G.S., Gautam P.B., Rai D.C., Kumari S. (2024). A Comprehensive Review on Donkey Milk and Its Products: Composition, Functionality and Processing Aspects. Food Chem. Adv..

[71] Faccia M., D’Alessandro A.G., Summer A., Hailu Y. (2020). Milk Products from Minor Dairy Species: A Review. Animals.

[72] Berezowski J., de Balogh K., Dórea F.C., Rüegg S., Broglia A., Gervelmeyer A., Kohnle L., European Food Safety Authority (EFSA) (2023). Prioritisation of Zoonotic Diseases for Coordinated Surveillance Systems under the One Health Approach for Cross-Border Pathogens That Threaten the Union. EFSA J. Eur. Food Saf. Auth..

[73] European Food Safety Authority (EFSA), European Centre for Disease Prevention and Control (ECDC) (2024). The European Union One Health 2023 Zoonoses Report. EFSA J..

[74] Plummer P.J., McClure J.T., Menzies P., Morley P.S., van den Brom R., van Metre D.C. (2018). Management of *Coxiella burnetii* Infection in Livestock Populations and the Associated Zoonotic Risk: A Consensus Statement. J. Vet. Intern. Med..

[75] Auriemma C., Lucibelli M.G., Bove F., Gallo A., de Carlo E., Martucciello A., Corrado F., Guarino A.G.G. Preliminary Survey on the Presence of *Coxiella burnetii* in Milk Samples from Donkeys Raised in Campania. Proceedings of the XIV Congresso Nazionale S.I.Di.L.V..

[76] Mora C., McKenzie T., Gaw I.M., Dean J.M., von Hammerstein H., Knudson T.A., Setter R.O., Smith C.Z., Webster K.M., Patz J.A. (2022). Over Half of Known Human Pathogenic Diseases Can Be Aggravated by Climate Change. Nat. Clim. Change.

[77] Bellinati L., Ceglie L., Kevenk T.O., Lucchese L., Natale A., Giacometti F., Alberghini L. Investigation on Q Fever agent in donkey milk. Proceedings of the EAVLD 2024—7th Congress of the European Association of Veterinary Laboratory Diagnosticians.

